# Cigarette, e-cigarette and waterpipe cognitions and use among university students in Guangzhou, China

**DOI:** 10.18332/tid/159171

**Published:** 2023-02-22

**Authors:** Sílvia Font-Mayolas, Mark J. M. Sullman, Jiawei D. Hughes, Maria-Eugenia Gras, Valentina Lucena Jurado, Fran Calvo

**Affiliations:** 1 Quality of Life Research Institute, Universitat de Girona, Girona, Spain; 2 Department of Social Sciences, University of Nicosia, Nicosia, Cyprus; 3 Department of Psychology, Drexel University, Philadelphia, United States; 4 Department of Psychology, University of Cordoba, Cordoba, Spain

**Keywords:** China, waterpipe, cigarettes, e-cigarettes, polytobacco use

## Abstract

**INTRODUCTION:**

There is currently little research on polytobacco use in China. The present study examined cognitions that predicted the use of cigarettes, e-cigarettes and waterpipes in a Chinese sample of students.

**METHODS:**

A convenience sample of 281 university students, obtained using snowball sampling, completed an online survey during the 2019–2020 academic year in Guangzhou, China.

**RESULTS:**

Men more strongly agreed, than women, with the possible advantages of using alternative nicotine and tobacco products, including: young people who smoke have more friends, smoking makes young people look cool, smoking makes young people feel more comfortable, smoking helps relieve stress, and it would be easy to quit. Factors significantly associated with regular cigarette use were the cognitions: ‘I would smoke if my best friend offered’, ‘Young people who use these products have more friends’, and ‘It would be easy to quit these products’ (global good classifications= 80.1%). In the case of waterpipes, agreement with the cognition: ‘The product helps people relieve stress’ was significantly associated with its use (global good classifications=80.1%). In the case of e-cigarettes, agreement with the cognitions: ‘I would smoke if my best friend offered’ and ‘It would be easy to quit using these products’ were significantly associated with the use of e-cigarettes (global good classifications=74.7%).

**CONCLUSIONS:**

The results highlight the need to develop prevention programs that prepare young Chinese people to resist social pressure from friends to use tobacco products. There is also evidence of the need to facilitate and disseminate rigorous scientific information among young people about the possible negative health effects of alternative tobacco products. There were also gender differences in the use of these products and in the cognitions towards their use, so it is important to take the gender perspective into account in the analysis of the results and when writing future questionnaire items.

## INTRODUCTION

Smoking is one of the leading causes of preventable premature death in the world^1^. Every year more than 1 million people die in China from diseases caused by smoking^1^. Furthermore, as a country at the center of the global tobacco epidemic, China faces a growing burden of tobacco-related health problems if there is no decrease in tobacco use^2^. In terms of prevalence, the 2018 Global Adult Tobacco Survey (GATS) found that 26.6% of the Chinese population aged ≥15 years were current cigarette smokers (50.5% men and 2.1% female) and 23.2% (44.4% men and 1.6% female)^3^ were current daily smokers.

Alternative nicotine and tobacco products (ANTP), such as e-cigarettes and waterpipes (also known as shisha, narghile, or hookah), have increased in popularity in several regions of the world^4^. In China, the prevalence of e-cigarettes has been increasing^5,6^, with the 2018 GATS reporting that 0.9% (1.6% men and 0.1% women) were current e-cigarette users^3^.

There is limited evidence about the prevalence of waterpipe use in China, but the World Health Organization (WHO)^7^ has suggested that in the South-East Asian Region there are a growing number of waterpipe bars and restaurants which young people primarily frequent. According to the 2018 GATS^8^, the prevalence of waterpipe use in China was 0.4%^3^. Furthermore, a survey (2010–2012) of the general population in South-West China reported that 8.5% also smoked waterpipes among current smokers^9^.

Although users of waterpipes and e-cigarettes commonly perceive these products as being healthy alternatives to regular cigarettes, the two products are not risk-free. The vapors from e-cigarettes contain several toxicants, some of which may be carcinogens^10^. Emerging evidence suggests that waterpipe tobacco also has long-term harmful health effects, similar to other forms of tobacco smoking^1^. Furthermore, several systematic reviews have investigated the relationship that e-cigarette use and waterpipe use have with various diseases and found waterpipe use to be associated with an increased burden of coronavirus (COVID-19) symptoms, as well as cardiovascular (e.g. atherosclerosis) and respiratory illnesses (e.g. lung cancer and chronic obstructive pulmonary disease)^11^. Furthermore, several studies have investigated the variety of substances people smoke from waterpipes, such as tobacco, herbal shisha, and marijuana^12^.

Polytobacco use can be defined as the concurrent use of regular cigarettes with other tobacco products, an emerging phenomenon among young adults in several regions of the world, including North America, Europe, and Asia^13,14^. In China, a study among smokers found that 15.9% were current poly users and of these 12.3% used one tobacco product in addition to regular cigarettes, and 2.5% used two tobacco products in addition to cigarettes^15^. Concerning health effects, polytobacco use has been shown to increase the risk of nicotine addiction and substance use disorders^16^.

Previous research has found that positive cognitions towards tobacco were associated with their use^17^. In a study of middle school students, e-cigarette use was associated with the belief that it helps people feel more comfortable in social situations and makes young people look more attractive^18^. Furthermore, an association was also found between positive cognitions towards waterpipes (i.e. making young people feel more comfortable and helping to relieve stress) and their use among adolescents^19^. Thinking that cigarettes are easy to quit has also been found to be associated with polytobacco use among high school students^20^. A similar association between positive cognitions towards e-cigarettes and waterpipes (i.e. relieving stress and looking cool) and their use was also found among young adults^21^. Moreover, the association between positive cognitions towards regular cigarettes and e-cigarette use (i.e. it would be easy to quit these products) has also been found among young adults who are polytobacco users^22^. However, it should be noted that most of these studies have been conducted in North America and Europe.

Research on e-cigarette and waterpipe use in China remains limited^23^, particularly concerning waterpipe use. Furthermore, having a better understanding of the specific characteristics of polytobacco users in China, a country with high tobacco use, will provide information that can be used to target public health interventions more accurately. This will mainly involve focusing on gender differences, in line with recommendations for conducting quality research^24^.

The objective of the present study was to analyze differences in the cognitions about regular cigarettes, e-cigarettes, and waterpipes by use, gender, and age, and to identify which cognitions were significantly associated with the use of these three products while controlling for gender and age.

## METHODS

### Study design and participants

The research design used in this cross-sectional study was based on previous research on polytobacco use^21^. The sample size was calculated so that the margin of error did not exceed 6%, with a confidence level of 95%. The online survey was shared through a campus network in Guangzhou, China, during the 2019–2020 academic year. A snowball sampling approach was also used, as students were asked to forward the survey to other university students studying in Guangzhou. Participation was entirely voluntary and without any form of inducement. A convenience sample of 281 university students completed the survey.

As Mandarin was the language of instruction, two native speakers of Mandarin and English used the forward-backward procedure to translate the survey into Mandarin. The inclusion criteria were: aged ≥18 years, the ability to read and understand Mandarin, and attending a university in the province of Guangzhou. Participants were self-selected into the study and provided informed consent. This study was approved by the Social Science Ethics Research Board (SSERB 0054) at the University of Nicosia, Cyprus.

### Measures of demographics, tobacco use, and cognitions

Demographics included the respondents’ gender, age, and ethnicity. Regarding tobacco use, participants were asked to report: ‘How frequently have you smoked regular cigarettes during the past 30 days?’. The same question was asked about e-cigarettes and waterpipes, and response options were ‘never’, ‘occasionally’, ‘once a week’, ‘more than once a week, but not every day’, and ‘every day’. Participants who reported ‘never’ using the three products (regular cigarettes, e-cigarettes and waterpipes) were classified as ‘non-users’, and those who answered ‘occasionally’, ‘once a week’, ‘more than a week, but not every day’ or ‘every day’ were classified as ‘current users’. Furthermore, participants who reported having smoked e-cigarettes over the past 30 days were asked whether the e-cigarettes were: ‘with nicotine’, ‘without nicotine’ or ‘with and without nicotine’. Likewise, participants who reported having used a waterpipe over the past 30 days were also asked whether the waterpipe was: ‘with tobacco’, ‘with non-tobacco or herbal shisha’, and ‘with marijuana or hashish’^25^.

*Tobacco use cognitions*

The cognitions associated with tobacco use were based upon previous work by Barnett and Livingston^26^. The questions were: ‘I would smoke if my best friend offered’, ‘young people who use these products have more friends’, ‘the product makes young people look cool’, ‘the product makes young people feel more comfortable’, ‘the product helps to relieve stress’, and ‘it would be easy to quit these products’. Participants were asked to report how likely they were to endorse each of the six cognitions for each tobacco product (cigarettes, e-cigarettes, and waterpipes) using the following response options: ‘definitely yes’, ‘probably yes’, ‘probably no’ and ‘definitely no’.

### Statistical analysis

Chi-squared tests were used to compare groups. When there were more than two groups, *post hoc* comparisons were used to compare subgroups. Effect sizes were computed using the phi coefficient. Fisher’s exact test was used when expected frequencies were <5. Three binomial logistic regressions were used to identify which cognitions were significantly associated with the use of each product (regular cigarettes, e-cigarettes, and waterpipes), controlling for age and gender. For these analyses, participants were divided into ‘non-users’ and ‘current users’, as defined above. The categories ‘definitely yes’ and ‘probably yes’ were combined, and so were ‘definitely no’ and ‘probably no’, for the data analysis. Furthermore, participants were divided into two age groups (18–24 and 25–44 years) for all analyses^27^. All analyses were performed using SPSS v23 (IBM Corp, Armonk, NY).

## RESULTS

The sample comprised 281 participants, 53.7% men, mean age 26.4 ± 6.13 years (range: 18–44), all of whom reported that they were Chinese.

### Tobacco use in the past 30 days

Table 1 shows the prevalence of using regular cigarettes, e-cigarettes, and waterpipes over the past 30 days, by age and gender. The proportion of participants who reported using each product over the past 30 days was: 42.7% regular cigarettes, 32.4% e-cigarettes, and 31.3% waterpipes. Significantly more men than women had consumed regular cigarettes [52.3% vs 31.5%; χ^2^(1)=12.3; p<0.001; φ=0.21] and e-cigarettes [40.4% vs 23.1%; χ^2^(1)=9.6; p=0.002, φ=0.19] over the last 30 days. However, although the trend was the same for waterpipe consumption, this difference was not statistically significant [35.8% vs 26.2%; χ^2^(1)=3.0; p=0.08].

**Table 1:** Percentage of regular cigarette, e-cigarette and waterpipe use in the past 30 days, by gender and age, among a sample of students^a^ during the 2019–2020 academic year in Guangzhou, China (N=281)

*Age (years)*	*Regular cigarettes*			*E-cigarettes*			*Waterpipes*		
	*Men (n=151)*	*Women (n=130)*	*χ^2^(1) (p) φ*	*Men (n=151)*	*Women (n=130)*	*χ^2^(1) (p) φ*	*Men (n=151)*	*Women (n=130)*	*χ^2^(1) (p) φ*
18–24 (n=129)	46.6	30.4	3.5 (0.06) -	32.9	26.8	0.6 (0.46) -	30.1	28.6	0.1 (0.85) -
25–44 (n=152)	57.7	32.4	9.8 (0.002) 0.25	47.4	20.3	12.5 (<0.001) 0.29	41.0	24.3	4.8 (0.03) 0.18
χ^2^(1) (p)	1.9 (0.17)	0.1 (0.80)		3.3 (0.08)	0.1 (0.38)		1.9 (0.16)	0.3 (0.59)	

a University students from Canton, China. φ: contingency coefficient (effect size)

More men than women reported consumption in both age groups, but these differences were only significant in the 25–44 age group for regular cigarettes, e-cigarettes and waterpipes.

Concerning polytobacco use, 52% of the participants had not used any of the three products over the last 30 days, 11% had only used one, 15.7% two, and 21.4% had used all three products. Table 2 shows polytobacco use by gender and age. There were significant differences in polytobacco use by gender. In both age groups, more women than men had not used any of the products over the last 30 days, and more men than women had used two products. There were no significant gender differences in using only one of the three products (Table 2). In addition, there were no significant differences by age group in men [χ^2^(3)=5.2; p=0.34] or women [χ^2^(3)=0.2; p=0.95].

**Table 2:** Tobacco use in the past 30 days and nicotine content, by age and gender, among students during the 2019–2020 academic year in Guangzhou, China (N=281)

*Tobacco use*	*18–24 years*		*χ^2^(3) (p) φ*	*25–44 years*		*χ^2^(3) (p) φ*
	Men* %	Women* %		*Men %*	*Women %*	
**Number of products used**						
None	45.2^a^	64.3^b^	10.3 (0.02) 0.28	35.9^a^	66.2^b^	14.5 (0.002) 0.31
One	15.1^a^	7.1^a^		12.8^a^	8.1^a^	
Two	24.7 ^a^	7.1^b^		20.5^a^	8.1^b^	
All three	15.1^a^	21.4^a^		30.8^a^	17.6^a^	
**E-cigarettes**			χ^2^(1) (p) φ			
Without nicotine	22.2	50.0	3.0* (0.08) -	67.9	64.3	0.1* (0.82) -
With and without nicotine	22.2	16.7		3.6	0.0	
With nicotine	55.6	33.7		28.6	35.7	
**Waterpipes**			Fisher test** p			Fisher test** p
With non-tobacco or herbal shisha	50.0	100	0.006	55.6	57.1	1
With tobacco	46.4	0.0		37.0	28.6	
With marijuana or hashish	3.6	0.0		7.4	14.3	

University students from Canton, China. φ: contingency coefficient (effect size). The different superscripted letters indicate significant differences between subgroups.

* To achieve compliance with the conditions of the chi-squared test, we combined the categories ‘with and without nicotine’ and ‘with nicotine’.

** We did not consider the n=5 participants that reported using waterpipe with marijuana, and used the Fisher exact test because some expected frequencies were <5.

### Nicotine content of e-cigarettes and waterpipe

Almost half (49.4%) of e-cigarette users reported using it without nicotine, 11.1% with and without nicotine, and 39.5% always included nicotine. Table 2 shows the percentage of e-cigarette users according to nicotine content by age and gender. Although more women than men reported using e-cigarettes without nicotine, these differences were not statistically significant in either age group. Amongst men, the use of e-cigarettes with nicotine was more frequent in the younger age group than in the older [χ^2^(1)=11.5; p=0.001; φ=0.46], but for women, there was no significant age group difference [χ^2^(1)=0.5; p=0.46].

Regarding waterpipe users, 59.8% reported using it with non-tobacco or herbal shisha, 34.1% with tobacco, and 6.1% with marijuana or hashish. Split by gender and age group, in the younger age group all women reported using waterpipes with non-tobacco or herbal shisha, but only five out of ten men reported this type of use. This difference was statistically significant. In the older age group, there were no significant differences between men and women (Table 2). Furthermore, there were no significant differences by age group in the use of tobacco with waterpipes in men [χ^2^(1)= 0.4; p=0.55] or women [ Fisher exact test : p=0.09].

### Tobacco use cognitions

Table 3 shows the percentage of participants who answered ‘probably or definitely yes’ to each cognition by product type, gender, and age group. For all three products, significantly more users than non-users thought that they would smoke if their best friend offered, that young people who use this product have more friends, that the product makes young people look cool and feel more comfortable and that it would be easy to quit the product. The exception to this was for the cognition that the product helps people relieve stress, where there were significant differences between regular cigarettes and e-cigarettes, but not for waterpipes. Furthermore, around 4 out of 10 non-users thought these products helped relieve stress.

**Table 3:** Tobacco product use cognitions (percentage of answers ‘probably yes or definitely yes’) according to use status, gender and age group, among students during the 2019–2020 academic year in Guangzhou, China (N=281)

*Tobacco products*	*Users*	*Non-users*	*χ^2^(1) (p) φ*	*Men*	*Women*	*χ^2^(1) (p) φ*	*18–24 years*	*25–44 years*	*χ^2^(1) (p) φ*
**Regular cigarettes**									
I would smoke if my best friend offered	69.2	14.9	85.8 (<0.001) 0.55	47.7	26.9	12.8 (<0.001) 0.21	36.4	39.5	0.3 (0.60) -
Young people who use regular cigarettes have more friends	51.7	19.9	31.2 (<0.001) 0.33	39.7	26.2	5.8 (0.02) 0.14	35.7	31.6	0.5 (0.47) -
Regular cigarettes make young people look cool	44.2	18.6	21.5 (<0.001) 0.28	37.7	20.0	10.6 (0.001) 0.19	32.6	27.0	1.0 (0.30) -
Regular cigarettes make young people feel more comfortable	42.5	22.4	13.1 (<0.001) 0.22	37.7	23.1	7.0 (0.008) 0.16	29.5	32.2	0.3 (0.62) -
Regular cigarettes help relieve people's stress	58.3	40.4	8.9 (0.003) 0.18	53.6	41.5	4.1 (0.04) 0.12	50.4	46.1	0.5 (0.47) -
It would be easy to quit regular cigarettes	53.3	14.9	47.2 (<0.001) 0.41	39.7	21.5	10.8 (0.001) 0.19	29.5	32.9	0.4 (0.54) -
**E-cigarettes**									
I would smoke if my best friend offered	78.0	20.0	87.2 (<0.001) 0.49	50.3	25.4	18.3 (<0.0001) 0.25	39.5	38.2	0.1 (0.81) -
Young people who use e-cigarettes have more friends	44.0	24.2	11.3 (0.001) 0.20	31.1	30.0	0.1 (0.84) -	30.2	30.9	0.02 (0.90) -
E-cigarettes make young people look cool	48.4	27.9	11.4 (0.001) 0.20	36.4	32.3	0.5 (0.47) -	34.1	34.9	0.02 (0.89) -
E-cigarettes make young people feel more comfortable	54.9	28.4	18.6 (<0.001) 0.25	43.0	30.0	5.1 (0.02) 0.13	35.7	38.2	0.2 (0.67) -
E-cigarettes help relieve people's stress	58.2	36.3	12.0 (0.001) 0.20	46.4	40.0	1.1 (0.28) -	42.6	44.1	0.1 (0.81) -
It would be easy to quit e-cigarettes	62.6	23.2	41.7 (<0.001) 0.36	44.4	26.2	10.1 (0.002) 0.19	37.2	34.9	0.2 (0.68) -
**Waterpipes**									
I would smoke if my best friend offered	59.1	24.4	31.9 (<0.001) 0.32	44.4	24.6	11.9 (<0.001) 0.20	36.4	34.2	0.2 (0.70) -
Young people who use waterpipes have more friends	56.8	18.7	41.5 (<0.001) 0.36	38.4	21.5	9.4 (0.002) 0.16	29.5	31.6	0.2 (0.70) -
Waterpipes make young people look cool	43.2	25.4	9.0 (0.003) 0.18	37.7	23.1	7.0 (0.008) 0.16	28.7	32.9	0.6 (0.45) -
Waterpipes makes young people feel more comfortable	48.9	27.5	12.3 (<0.001) 0.21	39.7	27.7	4.5 (0.03) 0.13	32.6	35.5	0.3 (0.60) -
Waterpipes help relieve people's stress	50.0	38.9	3.1 (0.08) -	44.4	40.0	0.5 (0.46) -	43.4	41.4	0.1 (0.74) -
It would be easy to quit waterpipes	60.2	23.8	35.1 (<0.001) 0.33	43.7	25.4	10.3 (0.001) 0.19	36.4	34.2	0.2 (0.70) -

University students from Canton, China. φ: contingency coefficient (effect size).

In terms of gender, more men than women answered ‘probably or definitely yes’ to all cognitions for all three substances. There were significant differences for five cognitions regarding regular cigarette and waterpipe consumption (except ‘the product helps relieve stress’ for waterpipes). In the case of e-cigarettes, there were significant differences only for the cognitions: ‘I would smoke if my best friend offered’, ‘The product makes young people feel more comfortable’ and ‘It would be easy to quit these products’. There were no significant differences in the cognitions between age groups (Table 3).

### Tobacco use models

Table 4 shows the results of three logistic regressions. The dependent variables were the use of each product and the independent variables were the six cognitions, controlled for age and gender. The factors significantly associated with regular cigarette use were the cognitions: ‘I would smoke if my best friend offered’, ‘Young people who use these products have more friends’ and ‘It would be easy to quit these products’. Participants who answered ‘probably or definitely yes’ to these cognitions were more likely to be users. The model correctly classified 73.3% of users and 85.1% of non-users (Global good classifications=80.1%).

**Table 4:** Factors significantly associated with tobacco products use, adjusted for age and gender, among students during the 2019–2020 academic year in Guangzhou, China (N=281)

*Tobacco products*	*p*	*AOR*	*95% CI*	*Nagelkerke R^2^ χ^2^ (p)*
**Regular cigarettes**				
Gender	0.13	1.56	0.87–2.85	0.42 103.4 (<0.001)
Age	0.94	1.00	0.96–1.05	
I would smoke if my best friend offered*	<0.001	7.12	3.71–13.67	
Young people who use regular cigarettes have more friends*	0.04	1.94	1.02–3.68	
It would be easy to quit regular cigarettes*	0.03	2.17	1.08–4.33	
**E-cigarettes**				
Gender	0.63	0.85	0.45–1.63	0.42 100.1 (<0.001)
Age	0.35	0.98	0.93–1.03	
I would smoke if my best friend offered*	<0.001	10.16	5.29–19.52	
It would be easy to quit e-cigarettes*	0.002	2.77	1.46–5.27	
**Waterpipes**				
Gender	0.78	0.92	0.50–1.67	0.29 63.8 (<0.001)
Age	0.86	0.99	0.95–1.04	
I would smoke if my best friend offered*	0.005	2.58	1.33–5.03	
Young people who use waterpipes have more friends*	<0.001	3.88	1.92–7.82	
Waterpipe use helps relieve people's stress*	0.02	2.38	1.17–5.51	
It would be easy to quit waterpipes*	0.002	2.83	1.46–4.48	

University students from Canton (China). AOR: adjusted odds ratio.

* Reference category: ‘Probably yes or definitely yes’.

In the case of waterpipes, agreement with the cognition: ‘The product helps relieve people’s stress’ was significantly associated with its use. Participants who answered ‘probably or definitely yes’ were more likely to be waterpipe users. The model correctly classified 67% of users and 86.3% of non-users (Global good classifications=80.1%).

Only the cognitions: ‘I would smoke if my best friend offered’ and ‘It would be easy to quit these products’ were associated with using e-cigarettes. Participants who answered ‘probably or definitely yes’ were more likely to be e-cigarette users. The model correctly classified 45.5% of users and 88.1 % of non-users (Global good classifications=74.7%).

## DISCUSSION

### Current tobacco use

Men used all three products (cigarettes, e-cigarettes, and waterpipes) more often than women. More than half (52.3%) of men and 31.5% of women had used regular cigarettes in the past 30 days. These results agree with the cigarette smoking rates among Chinese men reported in national surveys^1,3^. Furthermore, our findings support previous research, which has shown significant sex differences in regular cigarette use^3,15^, but we found a higher smoking rate among women than that reported in several previous studies^3,27^. These findings are worrying, considering the predicted number of deaths in China that will be caused by tobacco smoking in 2030 and 2050, if there is no decrease in this risky behavior^2^.

The present research found that 40.4% of men and 23.1% of women in our convenience sample had used e-cigarettes in the past 30 days. These results are higher than those found in previous studies among Chinese adults and adolescents^,5,6,28^. Perhaps the high percentage of e-cigarette users is related to the fact that e-cigarette use in China was unregulated until October 2019, when this product was removed from e-commerce websites, and strict legislative measures have not yet been developed^1,29^. Furthermore, this high rate may be due to the rapid introduction of e-cigarettes in China and the promotion of this nicotine delivery system as a ‘reduced risk’ product by the tobacco industry^3^. Nevertheless, the high rate of e-cigarette use is concerning, particularly as researchers have highlighted^23^ that many cigarette smokers could switch to this alternative nicotine delivery system, as well as its possible role in accelerating the tobacco epidemic in China.

The present study found that 35.8% of men and 26.2% of women had used a waterpipe in the past 30 days. As for e-cigarettes, these percentages are higher than those reported by previous studies among the Chinese population^5,8^. However, our data aligns with the report from WHO^7^, which highlighted the growing prevalence of waterpipe use among young people in China and several other regions of the world^4^. The role of waterpipe use in the progression to regular cigarette use^30^, and in the possible dissemination of nicotine use^23^, should be considered in future studies about the tobacco epidemic in China.

Concerning polytobacco use, the data confirmed that approximately half of the young people used two or three products concurrently, with 21.4% using all three products. Furthermore, men most often used two or more substances, while women mostly do not use any. The use of multiple tobacco products is becoming more common among young people. In a US study, Petersen et al.^31^ found that higher polytobacco use was significantly associated with higher cigarette consumption. The users of three or more products reported smoking significantly more cigarettes than those of one or two products, and users of two products smoked more than those who only used a single product. These findings suggest that the patterns of tobacco use have changed in recent years, highlighting the need for increased surveillance of the patterns of use among the different tobacco products^32^.

### E-cigarette and waterpipe nicotine content

One novel aspect of this research was to include questions about the nicotine content of the e-cigarettes and waterpipes used by this sample of Chinese users. The results showed that nearly half of all e-cigarette consumers reported consistently using this product to deliver nicotine and one in ten to sometimes deliver nicotine. With regard to waterpipes, nearly three in ten users included nicotine. Interestingly, a recent study about e-cigarette knowledge among Chinese university students found that fewer than half of the students knew whether e-cigarettes contained nicotine, and less than one-third identified e-cigarettes as tobacco products^28^. These findings show the importance of taking into account this variable in future studies about polytobacco use to more fully understand these risky behaviors.

### Cognitions about alternative nicotine and tobacco products

No differences were found in the cognitions about tobacco use by age group (18–24 years and 25–44 years). Instead, men agreed, more than women, with all cognitions studied across the three tobacco products investigated. In other words, this sample of Chinese men more strongly agreed, compared with women, with the following perceived advantages of using alternative nicotine and tobacco products: young people who use these products have more friends, using these products makes young people look cool, using these products makes young people feel more comfortable, using these products helps to relieve stress and it would be easy to quit using these products. The lower agreement with these positive cognitions among women could be related to their lower prevalence of use, as proposed by models which link attitudes and behavior^33^. Moreover, these findings make us reflect on whether the cognitions collected in the questionnaire adequately reflected how women think about these products, or whether it would be necessary to develop tools that are tailored for women^34^.

This study also found that higher consumption of e-cigarettes was related to those young people who thought that they would smoke or use electronic cigarettes if a friend offered it to them, that they would have more friends if they used e-cigarettes, and that it would be very easy to give up e-cigarettes. These results agree with another study carried out among Chinese secondary school students. Students with close friends who smoked were more likely to use e-cigarettes, and students who believed e-cigarettes could be addictive were less likely to use them^35^. Similarly, Fang et al.^28^ found that having friends and roommates who were e-cigarette users and holding more favorable attitudes towards e-cigarettes were associated with e-cigarette use among Chinese university students.

Our results also indicate the need to develop prevention programs in China that facilitate resistance to social pressure from friends. Furthermore, there is evidence of the need to facilitate and disseminate rigorous scientific information among young people regarding the possible adverse health consequences of alternative nicotine and tobacco products. As Lyu et al.^36^ propose, this dissemination must take into account the fact that the information from scientific forums is usually outside the reach of the general population of China. Instead, the population is usually informed via the media, which is frequently ambivalent towards these types of tobacco products. In order to reduce conflicting information, how to transfer information from scientific forums to these media is another pending focus of policymaking.

### Strengths and limitations

The present study contains several limitations. Firstly, this was a cross-sectional survey, so only associations between the variables can be reported. Furthermore, in terms of alternative nicotine and tobacco products, only e-cigarettes and waterpipes were investigated, so it is not possible to make conclusions about polytobacco use with other alternative products. Tobacco can be used in other formats, such as snuff, a smokeless method of using tobacco, which has been found to be used by 0.9% of the Chinese general population^3^. However, since this study was about smoking, this type of smokeless tobacco use was not included.

Another major limitation is that the study used a convenience sample that was self-selected, so the data may be different from Chinese university students in other regions. Furthermore, the research relied on self-report measures, which could be susceptible to potential biases such as social desirability bias.

Despite these limitations, the present study also has some strengths. The present research was conducted in a population with a high prevalence of tobacco use and there is very little previous research about alternative tobacco products, such as e-cigarettes and waterpipes. Furthermore, the present work offers new data about the increasing phenomenon of polytobacco use in this country. Another interesting element is the analysis of each product’s cognitions that are associated with tobacco use.

## CONCLUSIONS

The apparent increase in the use of e-cigarettes and waterpipes in the Chinese population highlights the need for increased surveillance of the patterns of use among the different tobacco products. The consumption of alternative tobacco products by friends and the positive cognitions towards this type of consumption should be considered when planning prevention campaigns. Future research should also address the content of e-cigarettes and waterpipes, specifically whether they contain nicotine or other psychoactive substances, to better assess their effects and consequences.

There were also gender differences in the use of these products and the cognitions towards their use, so it is important to take the gender perspective into account in the analysis of the results and when developing future questionnaire items.

## Data Availability

The data supporting this research are available from the authors on reasonable request.
